# Trabecular-Iris Circumference Volume in Open Angle Eyes Using Swept-Source Fourier Domain Anterior Segment Optical Coherence Tomography

**DOI:** 10.1155/2014/590978

**Published:** 2014-08-19

**Authors:** Mohammed Rigi, Lauren S. Blieden, Donna Nguyen, Alice Z. Chuang, Laura A. Baker, Nicholas P. Bell, David A. Lee, Kimberly A. Mankiewicz, Robert M. Feldman

**Affiliations:** ^1^Robert Cizik Eye Clinic, 6400 Fannin Street, Suite 1800, Houston, TX 77030, USA; ^2^Ruiz Department of Ophthalmology and Visual Science, The University of Texas Medical School at Houston, 6431 Fannin Street, MSB 7.024, Houston, TX 77030, USA; ^3^Glaucoma Service, South Texas Veterans Healthcare System, 7400 Merton Minter, San Antonio, TX 78229, USA

## Abstract

*Purpose*. To introduce a new anterior segment optical coherence tomography parameter, trabecular-iris circumference volume (TICV), which measures the integrated volume of the peripheral angle, and establish a reference range in normal, open angle eyes. *Methods*. One eye of each participant with open angles and a normal anterior segment was imaged using 3D mode by the CASIA SS-1000 (Tomey, Nagoya, Japan). Trabecular-iris space area (TISA) and TICV at 500 and 750 *µ*m were calculated. Analysis of covariance was performed to examine the effect of age and its interaction with spherical equivalent. *Results*. The study included 100 participants with a mean age of 50 (±15) years (range 20–79). TICV showed a normal distribution with a mean (±SD) value of 4.75 *µ*L (±2.30) for TICV500 and a mean (±SD) value of 8.90 *µ*L (±3.88) for TICV750. Overall, TICV showed an age-related reduction (*P* = 0.035). In addition, angle volume increased with increased myopia for all age groups, except for those older than 65 years. *Conclusions*. This study introduces a new parameter to measure peripheral angle volume, TICV, with age-adjusted normal ranges for open angle eyes. Further investigation is warranted to determine the clinical utility of this new parameter.

## 1. Introduction

Evaluation of the anterior chamber angle is essential to diagnose and manage eyes with glaucoma. Evaluating angle anatomy and monitoring changes in angle configuration after treatment, such as laser peripheral iridotomy (LPI) or lens extraction (LE), depend on the accuracy and precision of angle measurements. Several anterior segment optical coherence tomography (ASOCT) devices have been developed in the last decade [1] and have been shown to provide repeatable and reproducible measurements of the angle [2–5]. Although early generations of ASOCT instruments were able to assess angle measurements, their relatively slow scan rate did not capture adequate numbers of images in a feasible time frame, allowing for imaging of only 2 meridians (4 angles) in one scan.

**Figure 1:**
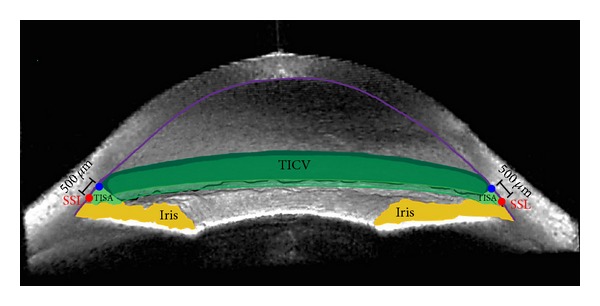
Trabecular-iris circumference volume (TICV). 3D anterior segment optical coherence tomography (ASOCT) image exhibiting TISA500 (light green space) and TICV500 (darker green spaces), along with the scleral spur landmark (red circle), iris (yellow), and cornea (violet line).

The commonly used quantitative measures to characterize angle structures are angle opening distance (AOD) and trabecular-iris space area (TISA) [6] (Figure 1). These measurements have been used to monitor changes in the anterior chamber angle morphology after LPI [7] or LE [8]. However, extrapolation of these measurements over the entire angle may be flawed because most ASOCT devices can only image 2 meridians in one scan.

The CASIA SS-1000 (Tomey Corporation, Nagoya, Japan) using Fourier domain (FD) swept-source technology can image 128 meridians (256 angles) in less than 5 seconds [1, 6]. This allows a 3D reconstruction of the anterior chamber angle and, therefore, more precise quantification of the angle structures. Trabecular-iris circumference volume (TICV; Figure 1) is the integrated volume of the peripheral angle derived from TISA taken at 256 locations in the angle. With any newly developed measurement, there is a need to establish the reference distribution. This study evaluates the normative distribution of TICV in open angle eyes using the CASIA SS-1000 FD ASOCT.

## 2. Participants and Methods

This prospective study was conducted at the Robert Cizik Eye Clinic of the Ruiz Department of Ophthalmology and Visual Science at The University of Texas Medical School at Houston. Institutional review board approval was obtained from The University of Texas Health Science Center Committee for the Protection of Human Subjects. All research was HIPAA compliant.

### 2.1. Participants

Participants 18 years of age or older were recruited between December 2012 and December 2013. One hundred and six participants distributed among 5 age groups (18–35, 36–45, 46–55, 56–65, and 66–79) met eligibility criteria. After obtaining informed consent, participants underwent slit lamp examination, intraocular pressure measurement, and gonioscopic examination performed by a glaucoma specialist (RMF, NPB, LSB, or DAL). Eyes with open angles (open to the ciliary body band or the scleral spur) were included. Lens grading was done using a scale of 0–4. Eyes were excluded if there was a history of intraocular surgery (such as LE or LPI), penetrating trauma, or any anterior segment abnormality that affected visualization of the angle or its measurements (i.e., significant corneal opacity). Participants were also excluded if they used any medication that may have affected angle anatomy within a month prior to imaging. When both eyes of the participant were eligible, one eye was randomly selected by coin flip. Although refraction was not performed, spherical equivalent data, where available, was recorded.

### 2.2. ASOCT Instrument

Instrumental details have been previously described [6]. For 3D image reconstruction, the CASIA SS-1000 obtains a series of 128 cross-sectional images (512 A-scans each) across the whole anterior chamber in less than 5 seconds. Each image dimension is 16 mm (length) × 16 mm (width) × 6 mm (depth).

### 2.3. Acquisition of ASOCT Images

Participant procedures for image acquisition have been previously described [6]. For 3D image reconstruction, eyes were scanned in 3D mode using the anterior analysis scan type with the autoalignment function.

### 2.4. Analysis of ASOCT Images

The images were exported from the CASIA SS-1000 and read by an experienced reader using customized software, Anterior Chamber Angle and Interpretation (ACAI, Houston, TX). The reader (AZC) was masked to the gonioscopy grade. The ACAI software divides 128 images into 8 panels, 16 images per panel (11.25 degrees between 2 consecutive angles). The reader marks the scleral spur landmarks (SSLs) [6] on each image in the first panel (this panel includes horizontal and vertical meridian images), and then the ACAI software automatically detects corneal edges and iris edges. If the edges of cornea and iris are not accurate, the reader manually adjusts intensity and, if not successful, manually adjusts the edge margins. Once the reader has completed and saved the interpreted result of the first panel, ACAI interpolates the SSLs in the remaining panels using the first panel result and detects edges. It should be noted that a previous study showed that 16 read images is sufficient to estimate TICV (within 5% mean absolute percent error) [9].

ACAI provides AOD and TISA at 500 and 750 *µ*m for each angle as well as radius (*R*), which is the distance from the midpoint of 2 SSLs to the centroid of each TISA. TICV500 and TICV750 are defined as bounded by the posterior corneal surface, anterior iris surface, scleral spur landmark ring, and 500 or 750 *μ*m centrally from scleral spur landmark ring, respectively. TICV500 and TICV750 were calculated using Pappus's centroid theorem formula:

(1) $$\begin{array}{c} \text{TICV}=2\pi \;\overset{256}{\underset{i=1\;}{\sum }}{\text{TISA}}_{i}\times \frac{R_{i}}{256}. \end{array}$$

### 2.5. Statistical Analysis

Demographics were summarized by mean and standard deviation (SD) for continuous variables or by frequency (%) for discrete variables. TISA at each quadrant (nasal, temporal, superior, and inferior) was summarized for all eyes and each age group. Comparing TISA among quadrants was performed using a mixed effect model. In this model, eye was the random effect, and quadrant was the fixed effect.

Histograms for TICV500 and TICV750 were plotted, as well as descriptive summary statistics, including mean, SD, median, range, and 2.5 and 97.5 percentile. Normality testing was performed to investigate whether TICV was normally distributed. Linearity between TICV500 and TICV750 was examined using regression analysis. TICV was summarized for each age group and compared using one-way analysis of variance (ANOVA). Mean and standard deviation of TICV measurements for each gonioscopic grade were calculated and compared using the two-sample* t*-test for validation. Furthermore, stepwise regression analysis was used to investigate the factors that affected TICV. The factors investigated were age, gonioscopic grade, gender, race, IOP, presence or absence of open angle glaucoma/suspect, lens grade (0 to 4, with 0.5 = trace), and spherical equivalent (sphere +1/2 cylinder). Analysis of covariance was used to compare TICV among age groups after adjusting for spherical equivalent.

All statistical analyses were performed using SAS for Windows v9.3 (SAS, Inc., Cary, NC). *P* < 0.05 was considered statistically significant for all comparisons.

## 3. Results

A total of 106 eyes of 106 participants were recruited. There were approximately 21 participants in each of the 5 age groups: 18–35, 36–45, 46–55, 56–65, and 65–79 years. Six eyes (5.7%) were excluded due to poor image quality, leaving a total of 100 eyes included in the study. Of those, 61% (61 eyes) were women. The mean (±SD) age was 50 (±15) years (range 20–79 years). Forty-three (43%) were right eyes. The study included 55 White (55%), 25 Black (25%), 10 Hispanic (10%), and 10 Asian (10%) participants. Gonioscopic findings included 58 eyes (58%) open to the ciliary body band, 41 eyes (41%) open to the scleral spur, and one eye (1%) open to the posterior trabecular meshwork. Thirteen eyes (13%) had primary open angle glaucoma without visible structural abnormalities. Two eyes (2%) had undergone laser-assisted in situ keratomileusis (LASIK) in the past. Forty-nine eyes (51%) showed presence of cataract. Eighty-eight (88%) eyes had documented spherical equivalent data. Baseline ocular characteristics are given in Table 1.

### 3.1. Trabecular-Iris Space Area

The cross-sectional iridocorneal angle parameters for all eyes and for each age group are summarized in Table 2. TISA500 and TISA750 were significantly smaller superiorly than in the other quadrants (*P* < 0.0001 for both TISA500 and TISA750). Differences between the other quadrants were not found to be statistically significant. The linear correlations between TISA500 and TISA750 were *R* ^2^ > 0.96, and the slopes ranged from 1.56 (nasal) to 1.70 (superior).

### 3.2. Trabecular-Iris Circumference Volume

**Figure 2:**
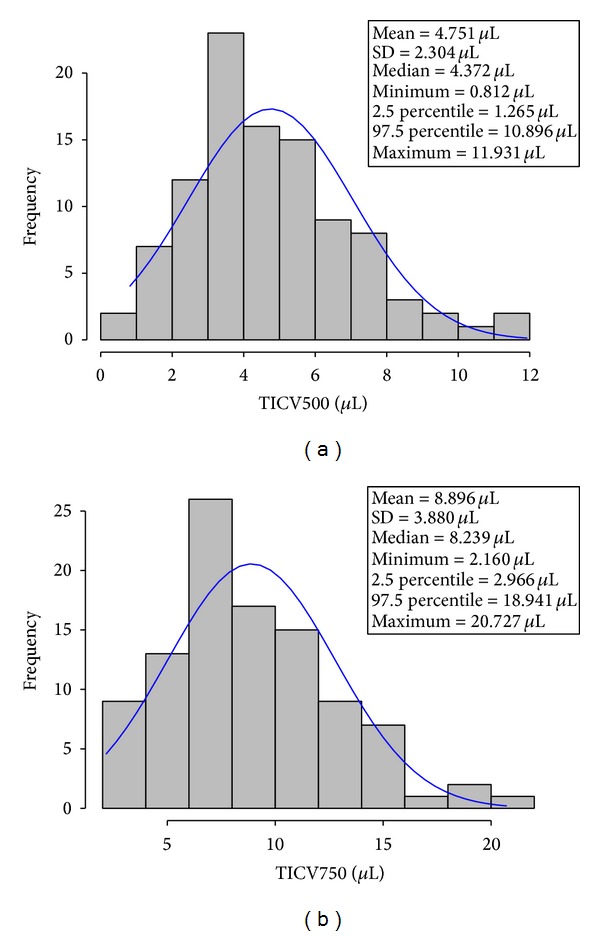
(a) Histogram and estimated density function for TICV500. (b) Histogram and estimated density function for TICV750.

**Figure 3:**
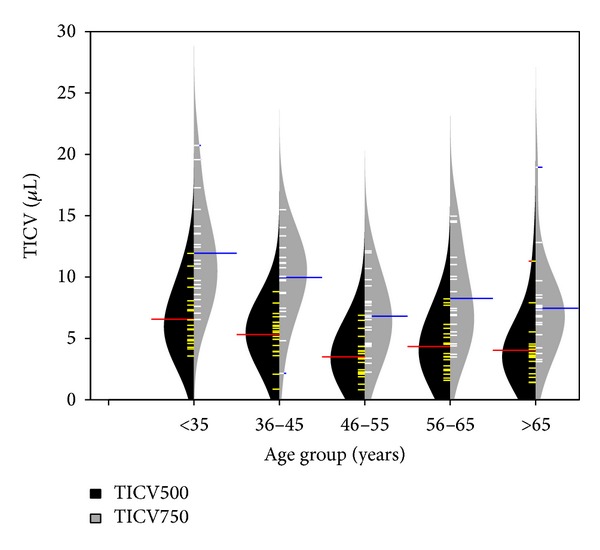
Observed TICV500 (yellow) and TICV750 (white), estimated normal density functions (black for TICV500 and grey for TICV750), as well as means (red for TICV500 and blue for TICV750) for each age group.

Figures 2 and 3 show the distribution of TICV500 and TICV750. The summary statistics for TICV500 and TICV750 are shown in Table 2. The means (±SD) were 4.751 *µ*L (±2.304) and 8.896 *µ*L (±3.880) for TICV500 and TICV750, respectively. TICV500 was normally distributed (*P* = 0.0873, Kolmogorov-Smirnov normality test), but TICV750 was not (*P* = 0.0385, slightly skewed to the right). A linear relationship, *R* ^2^ = 0.99, between TICV750 and TICV500 was observed, and the slope was 1.67. The mean (±SD) TICV500 was 3.246 *µ*L (±1.761) for eyes open gonioscopically to the scleral spur and 5.841 *µ*L (±2.028) for eyes open to the ciliary body band (*P* < 0.0001). The mean (±SD) TICV750 was 6.337 *µ*L (±5.407) for eyes open to the scleral spur and 10.749 *µ*L (±9.860) for eyes open to the ciliary body band (*P* < 0.0001). It should be noted that one eye gonioscopically open to the posterior trabecular meshwork was included in the group of eyes open to the scleral spur.

The 46–55-year-old group showed the smallest TICV (mean (±SD) 3.5 *µ*L (± 1.6) for TICV500 and 6.8 *µ*L (±2.8) for TICV750), which was significantly different from 18–35 and 36–45 age groups (Table 2), but not significantly different from the 56–65 and 65–79 age groups (one-way ANOVA with* post hoc* Duncan multiple comparison).

Age (*P* = 0.03455), lens grade (*P* = 0.0170), and spherical equivalent (SPE; *P* = 0.0402) influenced TICV500, using stepwise regression analysis. TICV500 decreased with age at the mean (±SD) rate of −0.37 *µ*L (±0.17) per decade, after adjusting for lens grade and SPE. Adjusted TICV500 decreased at a mean (±SD) rate of −0.89 *µ*L (±0.37) per grade of lens. TICV500 increased at a mean (±SD) rate of 0.12 *µ*L ± 0.06 per diopter of myopia. Similar results were obtained for TICV750.

**Figure 4:**
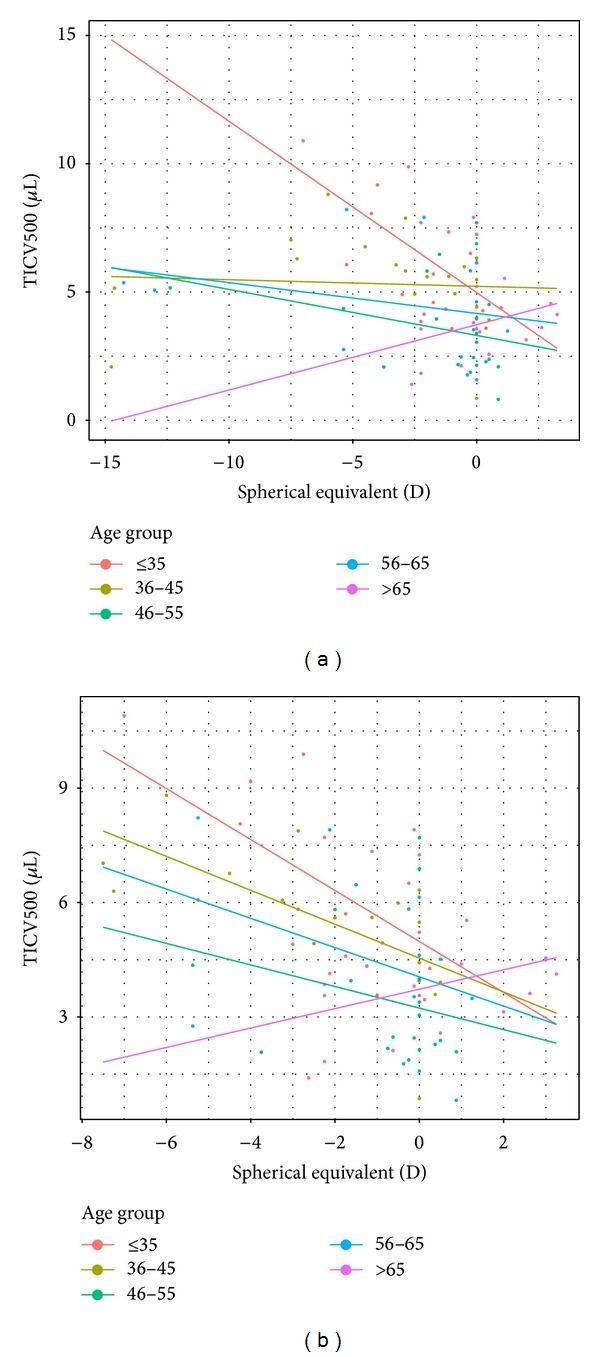
(a) TICV500 versus spherical equivalent scatter plot with estimated regression line for each group (with outliers). (b) TICV500 versus spherical equivalent scatter plot with estimated regression line for each group (without outliers).

In addition, to examine whether an interaction effect of age and SPE had any influence on TICV, a scatter plot of 88 eyes, where SPE data was evaluated, revealed that 5 eyes with high myopia (myopic refractive error > 12 diopters) skewed the estimates of slopes in the 36–45, 46–55, and 56–65 age groups (Figure 4(a)). After excluding those 5 highly myopic eyes, the results showed that the angle deepens as the degree of myopia increases (*P* = 0.0034 for TICV500 and *P* = 0.0012 for TICV750) for all groups except the oldest group (65–79 age group) (Figure 4(b)).

## 4. Discussion

This is the first study reporting a novel quantitative parameter, trabecular-iris circumference volume (TICV), and it establishes initial normal, age-adjusted reference values for open angle eyes. We found that TICV500 decreased with age at a rate of −0.37 *µ*L per decade, after adjusting for lens grade and SPE. This correlates with anatomic findings previously reported using other measurement techniques [10, 11]. Age affected TICV750 in a similar fashion. TICV500 and TICV750 showed a linear correlation, which indicates that both are equally suitable to quantitatively describe peripheral angle volume.

Previously, anterior chamber depth (ACD) estimation has been used to infer peripheral angle volume. The relationship between central ACD and peripheral angle volume has not been established, because until now peripheral angle volume could not be measured. In glaucoma, the overall anterior chamber depth is probably not as clinically relevant as the configuration of the peripheral angle. Estimates that use anterior chamber depth or volume as a marker for peripheral angle configuration may not be sensitive enough to detect small but clinically significant differences in the peripheral angle volume. The strength of TICV lies in determining peripheral angle volume, which accounts for only 2–2.5% of the anterior chamber volume [10].

TICV appears to have a normal distribution, when considering all age groups as well as within each age group, except within the oldest group (>65 years). We observed a similar range of TICV500 in both men and women. The age group 46–55 showed the smallest TICV500, which was significantly different from younger age groups (18–35 and 36–45), but not significantly different from older groups (56–65 and 65–79). The lowest volumes measured in the 46–55 age group may reflect subject selection bias, as eyes with progressively larger lenses causing clinically significant angle narrowing in the older age groups would be more likely to have undergone LE for vision reasons and not have been included in our study population (as pseudophakic eyes were excluded). Alternatively, lens enlargement may peak in the 46–55 age group and remain stable thereafter. It is also possible that the lens continues to enlarge but not in an anterior direction.

Overall, the results showed that TICV increased as the degree of myopia increased for all groups, except in the 65–79 group. This likely reflects the etiology of myopia in younger versus older age groups. In general, myopia in the former is typically caused by longer axial length or steeper corneal curvatures and in the latter by increasing lens power (cataract formation). This finding is also consistent with the selection bias mentioned above in that, as the population gets older, they are more likely to have visually significant cataracts that would undergo extraction, excluding them from our study population. It should be noted that the SPE was taken from habitual refractions, which may have overestimated myopia in younger patients with a masking of latent hyperopes prior to age 46.

We also observed that TISA in the superior angle was significantly smaller than other quadrants in all eyes (*P* < 0.0001). These results concur with the earlier studies showing that superior angle is the narrowest [3, 6]. We did not find a statistically significant difference between TISA measurements in the other quadrants.

There are several limitations to this study. Our results may not extrapolate to patients with anterior segment abnormalities or pseudophakia because this population was not included in our study. Also, we did not initially consider the effect that spherical equivalent would have on TICV. To better assess this relationship, further investigation is required. This sample may not be representative of the population as participants were recruited from patients, family members, and staff of a tertiary eye clinic. Results may not be generalizable outside of our inclusion and exclusion criteria.

In summary, this study describes a novel quantitative parameter, TICV, for measuring the peripheral angle and establishes a preliminary normal range of age-adjusted values. The reference range may require refinement adjusting for spherical equivalent. This deserves further study. With the introduction of normal values in open angle patients, further investigation is warranted to determine the clinical utility of this new parameter.

## Figures and Tables

**Table 1:** Baseline ocular characteristics.

Ocular Characteristics	Statistics
Iris Color, *N* (%)	
Blue	22 (22%)
Brown	70 (70%)
Hazel	8 (8%)
Cornea, *N* (%)	
Normal	88 (88%)
PEK	8 (8%)
Others (EBMD, KP)	4 (4%)
Presence of cataract, *N* normal (%)^1^	49 (51%)
Glaucoma, *N* (%)^2^	
Normal	60 (61%)
POAG suspect	26 (26%)
POAG	13 (13%)
IOP, mm Hg (±SD)^2^	14.94 (±3.04)
Number of IOP-lowering Medications, *N* (%)^2^	
0	81 (81%)
1	11 (11%)
2	6 (6%)
3	2 (2%)
Gonioscopy, *N* (%)	
Open to posterior trabecular meshwork	1 (1%)
Open to scleral spur	41 (41%)
Open to ciliary body band	58 (58%)
Spherical Equivalent, *D* (±SD)^3^	−1.93 (±3.64)

^1^3 missing data points.

^2^1 missing data point.

^3^12 missing data points.

PEK = punctuate epithelial keratopathy; EBMD = epithelial basement membrane dystrophy; KP = keratic precipitates; IOP = intraocular pressure; POAG = primary open angle glaucoma.

**Table 2:** Angle measurements [Mean (SD)] for all eyes and for eyes in each age group.

	Age (years)					
	All (*N* = 100)	≤35 (*N* = 20)	36–45 (*N* = 21)	46–55 (*N* = 21)	56–65 (*N* = 19)	>65 (*N* = 19)
TISA500 (mm^2^)						
Temporal	0.153 (0.070)	0.201 (0.074)	0.168 (0.057)	0.119 (0.044)	0.147 (0.080)	0.127 (0.061)
Nasal	0.160 (0.091)	0.208 (0.097)	0.172 (0.065)	0.122 (0.071)	0.154 (0.081)	0.143 (0.117)
Superior	0.109 (0.066)	0.158 (0.061)	0.130 (0.064)	0.073 (0.048)	0.092 (0.055)	0.090 (0.065)
Inferior	0.148 (0.080)	0.214 (0.081)	0.162 (0.065)	0.107 (0.066)	0.131 (0.082)	0.126 (0.064)
TISA750 (mm^2^)						
Temporal	0.286 (0.115)	0.369 (0.125)	0.313 (0.094)	0.227 (0.075)	0.280 (0.128)	0.239 (0.095)
Nasal	0.296 (0.144)	0.378 (0.152)	0.321 (0.113)	0.236 (0.117)	0.288 (0.146)	0.254 (0.159)
Superior	0.221 (0.113)	0.306 (0.105)	0.260 (0.113)	0.161 (0.083)	0.196 (0.101)	0.179 (0.100)
Inferior	0.281 (0.136)	0.391 (0.136)	0.311 (0.115)	0.212 (0.114)	0.253 (0.140)	0.235 (0.098)
TICV500 (*μ*L)	4.751 (2.304)	6.568 (2.432)	5.309 (1.810)	3.491 (1.640)	4.339 (2.085)	4.028 (2.316)
TICV750 (*μ*L)	8.896 (3.880)	11.934 (4.018)	9.966 (3.113)	6.808 (2.826)	8.257 (3.701)	7.461 (3.627)

TISA = trabecular-iris surface area; TICV = trabecular-iris circumference volume.
